# Why Men Rape: Perspectives From Incarcerated Rapists in a KwaZulu-Natal Prison, South Africa

**DOI:** 10.3389/fpsyg.2022.805289

**Published:** 2022-07-04

**Authors:** Lindokuhle Blessing Ngubane, Jani Nöthling, Relebohile Moletsane, Abigail Wilkinson, Lihle Qulu

**Affiliations:** ^1^School of Laboratory Medicine and Medical Sciences, College of Health Science, University of KwaZulu-Natal, Westville, South Africa; ^2^Gender and Health Research Unit, South African Medical Research Council, Cape Town, South Africa; ^3^Department of Psychiatry, Faculty of Medicine and Health Sciences, Stellenbosch University, Stellenbosch, South Africa; ^4^Genomics of Brain Disorders Research Unit, Faculty of Medicine and Health, South African Medical Research Council, Stellenbosch University, Cape Town, South Africa; ^5^School of Education, College of Humanities, University of KwaZulu-Natal, Durban, South Africa; ^6^School of Applied Human Sciences, University of KwaZulu-Natal, Pietermaritzburg, South Africa; ^7^Division of Medical Physiology, Faculty of Medicine and Health Sciences, Stellenbosch University, Cape Town, South Africa

**Keywords:** incarcerated rapist men, childhood trauma and adversity, rapist behavior, rape, recidivism

## Abstract

Sexual offending is a global problem but is particularly prevalent on the African continent and in South Africa. Childhood experiences related to abuse, alcohol use, and criminal activities in the household and community has been associated with an increased risk for violence perpetration in adulthood. Less is known about sexual violence perpetration, especially in the South African context. In this study, the experiences of incarcerated male perpetrators of rape in South Africa are investigated along with the collective social context and individual childhood experiences that potentially contribute to rape perpetration. Eighteen male perpetrators of rape who were inmates at Westville Correctional Services in KwaZulu Natal, South Africa, were interviewed. The semi-structured in-depth qualitative interviews were transcribed, coded and annotated using an interpretive paradigm and thematic analysis approach. Five main themes emerged from the research and included (1) childhood trauma and adverse events, e.g., an absent father, being raised without parents, exposure to criminal or violent behavior, physical abuse, sexual abuse and poverty, (2) understanding rape, e.g., rape as sex by force and without consent, rape as a violent act, rape as sex with a minor, myths about rape (3) substance abuse, e.g., history of alcohol and drug use, and intoxication during rape perpetration, (4) gender roles and avoiding responsibility, e.g., victim blaming, rape as male prerogative, transactional sex, being framed or set-up, ignoring an ancestral call and (5) recidivism. The findings revealed that all rape perpetrators were exposed to at least one childhood trauma type. Family and community violence and criminality was common. Most participants avoided taking responsibility for their actions and blamed the victim and recidivism/prior convictions were often reported. The findings demonstrate the complex personality dynamic involved in the cycle of abuse and the evolution of criminal behavior, starting as a victim and ending as a perpetrator. The findings also highlight the need for interventions aimed at reducing childhood trauma exposure and improving the social and relational context of those at risk for childhood neglect and abuse.

## Introduction

The South African Sexual Offences Act of 2007 defines rape as the genital, anal or oral penetration of the victim (without his/her consent) by the perpetrator. Penetration by the perpetrator includes genital, digital and object-penetration. Adult rape can take on many forms, e.g., acquaintance rape also known as date rape (perpetrator is known to the victim), stranger rape (perpetrator not known to victim), spousal rape (perpetrator is a marital partner), compelled rape (when the perpetrator forces the victims to have sex with each other), gang rape (multiple perpetrators) and corrective rape (rape as punishment for homosexuality). The underlying theme of rape, and what defines it as a criminal offense, is that the rape survivor does not consent to the act (Sexual Offences and Related Matters Amendment Act 32 of 2007, 2007).

Non-partner rape perpetration is particularly high in Africa (4.5 to 37.5%) compared to other continents, e.g., Australia (11.5–21.4%), South America (0.3–20.5%), Asia (0–20.2%), North America (0–16.9%) and Europe (0–15.7%) (Abrahams et al., 2014). South Africa has been labeled the rape capital of the world with an average of 42 559 cases being reported to the police each year and many more cases remaining unreported (Jewkes et al., 2013; South African Police Service [SAPS], 2021). Sexual offenses make up 8.5% of serious crimes committed annually of which 79% are cases of rape (South African Police Service [SAPS], 2020). While sexual offenses are not exclusively committed by men, men are the predominant perpetrators of sexual offenses targeted toward women and children (Yapp and Quayle, 2018). Intimate partner rape (husband, boyfriend or life partner) is more prevalent than non-partner rape (stranger, family member, relative, friend, neighbor, colleague or acquaintance) but is less commonly reported and is associated with a low conviction rate (Abrahams et al., 2014). In general, about a third of rape offenses are committed by perpetrators known to the victim (South African Police Service [SAPS], 2020). Stranger rape is usually associated with more severe violence inflicted on the rape survivor, e.g., use of force and threatening or enacting violence using a weapon (Jewkes et al., 2013).

Sexual offenses have far reaching psychological and economic consequences. Victims of rape and sexual assault are at higher risk of developing adverse physical (e.g., sexually transmitted infections, reproductive challenges, cardiometabolic risk) and mental health outcomes (e.g., depression, suicidality, post-traumatic stress disorder, anxiety disorders) compared to victims of other trauma types (Elklit and Christiansen, 2010; Nickerson et al., 2013; Tiihonen Möller et al., 2014; Dworkin, 2020). Rape perpetration and victimization places a significant burden on the economy of South Africa with the medical and psychological consequences of rape estimated to cost between R28.4 billion and R42.4 billion per year, representing 0.9 and 1.3% of the gross domestic product (GDP), respectively (Khumalo et al., 2014). The costs involved in legal consultation, prosecution and incarceration is estimated to be R48.7 billion per year.

A combination of emotional, social and cognitive adversities have been implicated in deviant behavior (e.g., antisocial behavior and an impaired ability to be accountable for actions) and sexual offenses (Ward and Beech, 2006). Adverse outcomes are shaped by the individual’s social, cultural, physical and interpersonal environment which has an impact on their emotional state, ability to act appropriately, to control emotions, perceptions of reality and modeling memories related to socially acceptable behavior (Ward and Beech, 2006). An important social/environmental factor that shapes emotional and cognitive states in adulthood is childhood trauma exposure (Carr et al., 2013). Men exposed to severe childhood trauma, e.g., abuse, neglect, community violence exposure and adverse parenting styles, are more likely than non-exposed men to develop depression, post-traumatic stress disorders, anxiety disorders, poor cognitive functioning and impaired learning (Gunnar and Vazquez, 2001; Bilbo and Schwarz, 2012; Scoglio et al., 2021). They are also more likely to display violent, antisocial behavior and to become chronic perpetrators of rape (Fox et al., 2015; Piotrowska et al., 2015; Moffitt, 2018). Victimized children also often have more violence supportive attitudes especially when exposed to domestic violence as children along with widespread societal exposure and acceptance of violence against women (Debowska et al., 2021).

Alcohol use during childhood and adolescence is another factor that may disrupt development and result in emotional, social and cognitive deficits (De Bellis and Zisk, 2014). Onset of alcohol use at an early age increases susceptibility to alcohol addiction in later life (Ploj et al., 2003). Childhood trauma has been associated with an increased risk of alcohol abuse in adolescence and adulthood (Andersen and Teicher, 2009), younger age of rape perpetration and more frequent offenses (Altintas and Bilici, 2018). Excessive alcohol consumption is associated with increased aggression and likelihood to commit rape (Jewkes et al., 2009), mediated by poor decision making and an increased sex drive (Jewkes et al., 2003).

Social and contextual factors encouraging male entitlement, hyper-masculinity, toxic masculinity and unequal gender norms are other key factors that drive rape perpetration (Maneta et al., 2017; Selepe et al., 2020). Hyper-masculinity and toxic masculinity are associated with insecurities, hypersensitivity, distrust toward women and satisfaction obtained from controlling and dominating women (Malamuth et al., 1996). Rape perpetrators often describe rape as a way to exert power, dominance and control over women and use it indirectly as a tool to conceal insecurities (Selepe et al., 2020). Linked to this, male entitlement, where men believe that they have total control over women and their bodies, influences them to perpetrate sexual violence (Selepe et al., 2020). One study reported that male perpetrators believe that as men, they are entitled to sex with their female partners regardless of their consent, especially if they are married (Adinkrah, 2011).

While there are many studies investigating the social factors influencing male criminal behavior in general (Fox et al., 2015; Altintas and Bilici, 2018), less is known about the factors that shape sexual violence and rape perpetration specifically. In order to adequately address the issue of rape and sexual violence against women, research that focuses on the factors that influence male rape perpetration is needed. This may be of benefit to rehabilitation programmes and to determine factors associated with recidivism (Abramsky et al., 2014; Selepe et al., 2020). In this study we investigate the experiences of incarcerated male perpetrators of rape in South Africa along with the collective social context and individual childhood experiences that potentially contribute to rape perpetration.

## Methodology

### Design

We followed an exploratory design using an interpretive paradigm which suggests that reality is not singular or objective but involves an inter-subjective epistemology and ontology (Jackson, 2011). A qualitative approach was used to explore incarcerated men’s perspectives on why they have raped. This approach allows the understanding of various participant experiences and further gives insight into contextual and cultural factors that impede or enhance the efficacy and social/ecological validity of interventions (Leech and Onwuegbuzie, 2007). Numerous sexual violence research studies have been conducted globally, but there is a dearth on research focusing on incarcerated male perpetrators of rape. This is especially true in the South African context where rape perpetration is high and violence is normalized following nearly 50 years of institutionalized racial segregation, civil unrest and intergenerational trauma under the South African apartheid regime (Hirschauer, 2014).

### Participants and Setting

The study was conducted at Westville Correctional Services (prison) in KwaZulu-Natal, South Africa. Westville Correctional Services is one of the largest correctional facilities in South African and houses approximately 12500 offenders. Westville consists of five correctional centers housing (1) unsentenced offenders awaiting trial, (2) sentenced male maximum security offenders, (3) male short-to-medium security offenders, (4) sentenced youth offenders, and (5) sentenced female offenders. The prison system in South Africa is referred to as correctional services, given that there is a strong focus on rehabilitating offenders and reintegrating them into society once they have served their sentence. Various rehabilitation programmes are offered at Westville and generally focus on developing life skills, psychoeducation, diversion using sports and recreational activities, correcting sexual and aggressive behavior, drug and alcohol use education, promotion of social responsibility and the development of skills to encourage productivity and financial security (Singh, 2014).

For the purpose of this study we used purposive and convenience sampling to recruit male perpetrators of rape, housed in the sentenced short-to-medium security offenders center. We interviewed a total of 18 participants. All participants were sampled from the same correctional center and data from all participants were used in the analysis. The inclusion criteria were (1) male, (2) between 18 and 65 years, (3) convicted of any rape charge, and (4) incarcerated at Westville Prison in KwaZulu Natal. Participants were excluded if they were unable to understand and speak either isiZulu or English, and unable to consent to participating in the study (e.g., due to intellectually disability).

### Procedure

All men were recruited through the Head of Psychological Services, who provided them with the study information sheet that described the purpose and procedure of the study. Men who agreed to further contact were invited to meet the researcher (a male Master’s student with training in qualitative research methods). The researcher explained the study to potential participants and those interested in taking part completed the informed consent form with the researcher. All interviewees participated voluntarily and were informed that they could withdraw from the study at any time point without any adverse consequences to themselves. Semi-structured interviews were conducted by the researcher to obtain a rich narrative suitable for qualitative analysis. An interview guide with open-ended questions and tentative probes was used to elicit the participants’ subjective accounts of (1) their personal history and lifepath, (2) childhood history and family history of violence, (3) their perspectives on sexual behavior, (4) myths or beliefs related to sexual violence and rape, (5) their mentors or role models, (6) perspectives on criminal behavior and cultural contextualization, and (7) their religious and spiritual backgrounds. Participants were interviewed individually in a private consultation room with no guard present to ensure confidentiality. The interviews lasted between one hour to one and a half hours and continued until saturation point was reached. The interviews were audio-recorded with the written permission of participants. The researcher also took notes during the interviews.

The Department of Correctional Service in South Africa approved the study and allowed permission to conduct the research on their premises. The study was approved by the Biomedical Resource Ethics Committee (BREC) of the University of KwaZulu-Natal (BREC No. BE 129/19).

### Data Analysis

IsiZulu audio recordings were translated to English and all recordings were transcribed for analysis. The recordings were destroyed after transcription as was agreed upon with participants. Thematic analysis was used to analyze the data from the interview transcripts. Thematic analysis allows for the identification and description of themes with an intention to examine deeper meaning, individual perspectives, assumptions and knowledge of a particular subject in relation to the research question (Braun and Clarke, 2006). The transcripts were read extensively by four researchers who coded and annotated the data to identify underlying themes (Braun and Clarke, 2006). The themes were iteratively subjected to review and refinement during data analysis as per the six phases of thematic analysis described by Braun and Clarke (2006). The themes emerging from the narratives provided illustrates the collective experiences and perspectives of male perpetrators of rape.

## Results and Discussion

### Demographic Layout of the Sample

The sample consisted of eighteen men incarcerated for rape perpetration. The majority of the participants were Black (see the footnote 1 in Table 1), middle aged, heterosexual and single. Most participants were either unemployed, temporarily employed or supported by their parents/guardians at the time of committing the rape. Most were born and raised in rural areas with low or no education. Participants were sentenced to imprisonment, from 8 years to life (25 years). The majority of the victims were girls under 18 years of age. The majority of participants knew the victim (parents/guardian, partner, neighbor, relative or family friend) before the rape occurred. All participants were single assailant offenders for their convicted rape cases with no gang/group rape. Some participants and a few victims were under the influence of substances (alcohol/drugs) when the offenses were committed. Only a few participants used a weapon at the time of the offense. The demographic layout of the sample is presented in Tables 1, 2.

**TABLE 1:** Demographic information of the perpetrators and victims.

Participant identifier	Current age of perpetrator	Ethnicity of perpetrator^1^	Sexual orientation of perpetrator	Education level of perpetrator	Age at the time of perpetration	Relation-ship status at the time of perpetration	Employment status at the time of perpetration	Home location at the time of perpetration	Legal plea	Sentence (in years)	Victim’s age	Victim’s sex	Offender–victim relationship	Use of weapon during incident	Offender/Victim intoxicated during incident
P1	44	Indian	Homosexual	Primary	21	Single	Part-time	Township	Guilty	25	10	Male	Non-stranger	No	No
P2	46	White	Heterosexual	Secondary	34	Divorced	Part-time	Suburb	Not Guilty	18	15	Female	Non-stranger	No	Offender
P3	25	Black	Homosexual	Secondary	15	Single	Unemployed	Township	Not Guilty	20	8	Male	Non-stranger	No	No
P4	65	Indian	Heterosexual	Primary	21	Divorced	Unemployed	Township	Not Guilty	6	18	Female	Non-stranger	No	No
P5	25	Black	Heterosexual	Secondary	17	Single	Unemployed	Township	Not Guilty	15	16	Female	Stranger	Yes	No
P6	29	Black	Heterosexual	Secondary	27	Single	Part-time	Rural	Not Guilty	8	23	Female	Non-stranger	No	Both
P7	56	Black	Heterosexual	Primary	49	Married	Full-time	Rural	Not Guilty	18	12	Female	Non-stranger	No	No
P8	36	Colored	Heterosexual	Secondary	28	Single	Unemployed	Township	Guilty	17	Could not remember	Female	Non-stranger	Yes	Yes
P9	56	Black	Heterosexual	None	38	Married	Full-time	Rural	Not Guilty	25	14	Female	Non-stranger	No	No
P10	40	Black	Heterosexual	Secondary	31	Single	Full-time	Rural	Not Guilty	22	11	Female	Non-stranger	No	No
P11	61	Black	Heterosexual	Primary	48	Single	Full-time	Rural	Guilty	25	28	Female	Stranger	Yes	No
P12	51	Black	Heterosexual	Secondary	48	Single	Part-time	Rural	Not Guilty	17	12	Female	Non-stranger	No	No
P13	27	Black	Heterosexual	Secondary	23	Single	Unemployed	Township	Not Guilty	15	38	Female	Non-stranger	No	Both
P14	63	Black	Heterosexual	Secondary	48	Married	Full-time	Rural	Not Guilty	25	14	Female	Non-stranger	No	No
P15	48	Black	Heterosexual	Secondary	34	Single	Full-time	Rural	Not Guilty	19	27	Female	Non-stranger	Yes	No
P16	37	Black	Heterosexual	Primary	27	Single	Part-time	Rural	Not Guilty	22	6	Female	Non-stranger	No	Offender
P17	29	Black	Heterosexual	Secondary	26	Single	Part-time	Rural	Not Guilty	17	27	Female	Non-stranger	No	Offender
P18	42	White	Heterosexual	Secondary	30	Married	Full-time	Suburb	Guilty	19	13	Female	Non-stranger	No	No

*^1^We acknowledge the problematic nature of the racial categorization reminiscent of the apartheid era. We use these categories to illustrate the influence of cultural norms and the gender inequality embedded in them in various communities and groups in South Africa.*

**TABLE 2:** Main themes and sub-themes associated with rape perpetration.

	Main theme	Sub-themes
Theme 1	Childhood trauma and adverse events	Absent father Orphaning (being raised without parents) Exposure to criminal or violent behavior Early physical abuse Early sexual abuse Family poverty
Theme 2	Understanding rape	Rape as sex by force and without consent Rape as a violent act Rape as sex with a minor Myths about rape
Theme 3	Substance abuse	History of alcohol and drug use Intoxication during rape perpetration
Theme 4	Gender roles and avoiding responsibility	Victim blaming Rape as male prerogative Transactional sex Being framed or “set-up” Ignoring an ancestral call
Theme 5	Recidivism	

### Emerging Themes

Five major themes were identified through our analysis, these include (1) childhood trauma and adverse events, (2) understanding rape, (3) substance abuse, (4) perceptions of gender roles and avoiding responsibility, and (5) recidivism. The themes and sub-themes are presented in Table 2.

#### Theme 1: Childhood Trauma and Adverse Events

The first theme “childhood trauma and adverse events” was the most common theme emerging from the findings. The majority of participants reported having been exposed to some sort of adverse experience during childhood and/or adolescence prior to being convicted of rape. The environmental exposure to childhood trauma may have influenced the development and behavior of participants and contributed to the way they perceive rape perpetration and victimization. The sub-themes that emerged were having an absent father, orphaning, exposure to criminal or violent behavior, early physical abuse, early sexual abuse and family poverty.

##### Absent Father

Most participants indicated that they grew up without a father or father figure in their childhood. Some participants stated that they never knew their father at all because he had died, while others indicated that their father was alive, but not present in their lives:

*My father wasn’t there; it was hurtful, and it was painful not to see my father being present. It was very painful to see other families, with my family not there (Participant 1).*

*My father passed away when I was maybe* 6 months [old]. *I was very small*. *I never grew up with my father*…*with their fighting I ended up staying with my granny. There is nothing I can say about him* (Participant 8).

##### Orphaning (Being Raised Without Parents)

Some participants reported that both of their parents were absent in their childhood and they grew up without a stable parent:

*When I was born, I was taken from my mother and placed in a home of safety where*.

*I grew up with my brothers and sisters and they was one* [were the ones] *that look* [looked] *after me, change my napkins* [nappies/diapers], *fed me and things like that* (Participant 1).

*My parents died when I was very young*…*I was raised by grandparents and* [we] *lived together* (Participant 9).

##### Exposure to Criminal or Violent Behavior

Participants reflected on childhood exposure to violence and witnessing family or community members being abused:

*My father stabbed my mother and got arrested* (Participant 16).

*I witnessed abuse not in my house but in other people’s house* [houses]…*like neighbors, I saw a man hitting his wife. A man gets drunk, fight* [fights] *with his wife, burns his wife with the oil, all sort things* (Participant 4).

Many participants reported having family members who have been arrested for interpersonal violence and other criminal activities:

*My brother was arrested because he assaulted a female and he crashed a car on someone. Another brother of mine, up until now I don’t know* [of] *him since 2005 where there was a teacher’s strike* (Participant 5).

*My father’s brother who was arrested when I was old* [older], *my father, my aunt’s child and my younger brother was* [were] *arrested* (Participant 1).

##### Early Physical Abuse

Some participants indicated that they had been victims of physical abuse during childhood, for example beatings with a sjambok^1^, or being forced into completing manual labor:

*A sjambok was used, they started beating me with it when I was 5. They poured water on me then beat me so that it will stick* (Participant 6).

*My stepmother came at* [to] *my father’s house around 04:00 and chased me away. I took my belongings, bathed in the river, got dressed and went to school*. *She* [stepmother] *would take me and hit me on the floor with* [on] *my body* (Participant 5).

*He put the chilies into my eyes, curry powder*…*and then in my*…*both eyes, then he put* [it] *on my mouth, he locked me in the room*…[I] *can still remember this. I got a good hiding from my father, they tied me to a tree*…*put sugar water* [on me], *I was naked, thick sugar water, brown sugar. He pastes it on me, he takes an ant, I can still remember, put an ant there. From one ant it came to many ants and I’m telling you the ants were biting me. I can’t forget, up to now, what happened to me that day* (Participant 4).

Some participant reported strong emotions as a result of punishment:

*You build up anger on* [in] *you and you swear in your mind, then you think what can I do to this person, it was like that. I had anger on* [in] *me. Anger to revenge that’s* [that what’s] *right* (Participant 4).

*There’s sort of hatred*…*you know when you* [you’re] *growing up as kids, you don’t understand why you were punished, because you think you are doing the good* [right] *things all the time* (Participant 10).

*I would be angry at her and sometimes wish she dies* [would die], *because she whipped me, and I was young at that time. I would feel she is abusing me* (Participant 13).

##### Early Sexual Abuse

Many participants shared their experience of being sexually abused during childhood and most often by a family member. One participant indicated that he was sexually abused at a young age by his mother and siblings:

*My sexual abuse started when I was* [a] *small child, I won’t say the age cause* [because] *I won’t know the age, but I was abuse* [abused] *by my mother, by my brothers and sisters* (Participant 1).

*I was taught by girls how to have sex; I became a laughingstock because I could not be turned on when they were on. They would play with it and they wanted me to sleep with my cousin. Even though it was considered playing, but it was very uncomfortable to* [for] *me* (Participant 6).

*My brother put me into* [onto] *sex when I was about 14 years. My brother found a girl for me*…*He taught me what do you do when you have a girlfriend*…*When we slept* [together], *it was my first time and it was her first time as well. We got stuck to each other and we could not separate. My brother saw that I am* [was] *trying to pull myself out of her, but I can’t* [couldn’t] *and I was crying*…*remember I don’t* [did not] *know anything. He pulled me out* (Participant 12).

##### Family Poverty

Most participants indicated that they grew up poor, with government social grants as a main source of income or their parent(s) earning minimum wage while working as a manual laborer or as a domestic worker:

*When I was small, I use* [used] *to look for food in the bin because there was no food at home, so it’s been like that most of my life* (Participant 1).

*After his* [father’s] *arrest, we had no income. It was hard, hard, hard. When I tell you hard, it was very hard* (Participant 4).

Many participants reported that they had to drop out of school to provide for themselves and their families:

*I have standard 8* [grade 10]. *I didn’t finish school because my mother was alone. When we went to high school, there was* [were] *3 of us, since I was older than the 2, it was clear that I should stop* [school] *so she can continue with the 2* (Participant 12).

*I was taken out of school to work. During the apartheid that’s how things were, the white man said my mother can’t stay in* [on] *the farm without anyone working for the farm. He wanted me to work, for my mother to have a place to stay. That’s how I stopped school even though I loved school* (Participant 7).

*I stopped at standard 6* [grade 8], *what I needed the most was education. Unfortunately, the elders were not educated, and they wanted us to herd the cows and didn’t* [not] *go to school. I started school at 16, at that time you are already stubborn*…. *It was painful, especially when I see other kids going to school. I would leave the cows alone and go to school, the cows would go to people’s field* [fields] *and I would be whipped for it. It was very painful, I really loved to study* (Participant 15).

The results related to childhood trauma and averse events support the findings of several prior studies that report a strong link between difficult childhood experiences and rape perpetration (Bendall, 2010; Jewkes et al., 2010; De Bellis and Zisk, 2014). All participants reported at least one form of early adversity, for example, an absent father, exposure to community violence, physical abuse, sexual abuse and poverty. Developmental theories of criminal behavior suggest that abuse and neglect has an especially prominent effect during the first 3 year of life when bonding and forming a secure attachment with a primary caregiver is of substantial importance in laying the foundation for socially acceptable behavior, driven by a reciprocal relationship of trust, safety, empathy and care (De Bellis and Zisk, 2014; Grady et al., 2021). When a caregiver neglects, abuses or acts inconsistently toward a child, the child is likely to display social withdrawal which may be considered a precursor to antisocial behavior (behavior that causes alarm or distress for others) often displayed by criminals and perpetrators of rape, e.g., lack of meaningful relationships, intimacy, empathy, guilt, remorse and morality as well as aggressive, impulsive and violent behavior (Johnson, 2019). The effect of childhood trauma generally accumulates in a dose-response manner with chronic and repeated trauma associated with an incremental increase is antisocial behavior (Cross et al., 2017; Lutz et al., 2017).

Poverty, educational challenges and absent or deceased parents further add to the burden of childhood trauma. South Africa has a high rate of HIV with approximately 13.7% of the population living with HIV and a high rate of concomitant tuberculosis (Karim et al., 2009; Thindwa et al., 2021). Prior to 2004 there were no treatments available for HIV in South Africa and an estimated 3 million South Africans lost their lives to HIV (Johnson et al., 2017). This resulted in many children losing their parents at a young age and an influx in single-parent or child-headed households (Richter and Desmond, 2008). Due to these difficult socio-economic circumstances many children were forced to abandon their education and resort to the streets to beg for food or work in order to take care of themselves, an ill parent or their younger siblings (Hartell and Chabilall, 2005). This leaves children vulnerable to becoming involved in criminal activities (e.g., stealing, prostitution) and becoming both victims and perpetrators of sexual abuse (Hartell and Chabilall, 2005; Cluver and Gardner, 2006). In the absence of parental care, there is also an absence of support which is a key protective factor against rape perpetration (Cluver and Gardner, 2006).

#### Theme 2: Understanding Rape

The second theme “understanding rape” highlights interviewees’ understandings of what rape is as well as the laws around what constitute rape and common myths associated with rape perpetration. Most participants had some understanding of rape, e.g., sex without consent and sex using coercion, force or violence. Other participants lacked a clear understanding of rape. Some participants did not mention consent, and a few did not understand the concept of statutory rape. A few participants were aware of common myths associated with rape and HIV/AIDS. This theme yielded five sub-themes, namely: rape as sex by force, rape as sex without consent, rape as a violent act, rape as sex with a minor and lastly myths about rape.

##### Rape as Sex by Force and Without Consent

When asked for a definition of rape, most participants explained that rape was sex by use of force and without consent, which indicates a basic understanding of rape:

*When you force yourself* [on] *to a woman, that’s what I understand* (Participant 10).

*It* [is] *said to be a forcible genital penetration without the consent* (Participant 5).

*I understand rape as taking a female by force without her consent* (Participant 8).

Some interviewees also understood that rape without the use of force still constitutes a criminal offense if the victim did not consent:

*Rape is something, for* [from] *my understanding, is where you have sex with a person, but it’s done without the person’s will* (Participant 1).

*I understand it as having sex without consent. The one being raped doesn’t enjoy its pleasure, it’s the rapist that enjoy the pleasure* (Participant 13).

*To me rape is anything with gender, it’s not against a female but without their consent, it’s that simple* (Participant 2).

##### Rape as a Violent Act

One participant described that rape is often violent and is an act against women:

*What I knew about rape was that a violent person will meet a female, beat her up, tear her underwear and have sex with her*…. *Also, for those with cars, they would grab a female and take her to their homes and have sex with her as she cries, that’s rape* (Participant 9).

##### Rape as Sex With a Minor

A lack of understanding of statutory rape emerged from the data as some participants expressed that the underage victims of their sexual deviant behavior agreed to a sexual act and they were unaware of the legal definition of statutory rape. For example, participant 10, indicated that the victim had no problem, the problem was with the parents:

*Although she was a child* [age 13], *no one was forced. She was not feeling bad until her parents saw the incident and arrested me, but her, she had no problem* (Participant 10).

Another participant reported that the underage victim of his sexually deviant behavior gave consent and that both of them did not view the act as rape:

*He [age 8] had no problem when I kissed him. I touched him nicely. He touched me, that means it’s all good. According to us, we had no problem, we did this in a* [the] *right way with a consent between us. No one thinks this is abuse, it never came to him* (Participant 3).

One participant did express understanding that rape includes sex with a minor:

*My understanding of rape is when a man forced himself to* [onto] *a woman or to a person underage* (Participant 16).

##### Myths About Rape

There was some awareness of myths associated with rape among the participants. The choice of words used by participants generally indicated that although they were aware of the myths, they did not necessarily believe them to be true. Some participants reported on myths related to HIV/AIDS and rape:

*To say if you sleep with a small child you won’t be infected of* [with] *HIV. I heard that if you sleep with a small child you get cured of HIV. That’s the only one I heard* (Participant 5).

*I’ve heard in the media about if you’ve raped a virgin, you’ll cure AIDS, all kind of rubbish like that* (Participant 18).

The legal definition of rape was generally well understood by the participants and is most likely the results of psychoeducation around sexual and aggressive behavior offered as part of the rehabilitation program to prisoners in Westville Correctional Services (Singh, 2014). A common myth, reminiscent of the period before 2004 when HIV denialism in South Africa resulted in a lack of treatment and high mortality rates, is that sex with a virgin will cure HIV infection (Leclerc-Madlala, 2002). An increase in HIV education and a decrease in HIV mortality after 2004 lead many to come to the conclusion that this is nearly a myth and should not be pursued as a cure for HIV (Sivelä, 2016). The myth was mentioned by many participants and acknowledged as a myth.

While rape of an adult women was well understood, rape of a minor was not. Some perpetrators of child rape described the act as consensual and did not view it as an offense. This behavior may be ascribed to the phenomenon where survivors of childhood abuse identify with the perpetrator and internalize the pleasurable experiences of the perpetrator (Maniglio, 2012; Minnaar, 2015; Moffitt, 2018). The act of child rape is generally minimized and rationalized in an effort by the perpetrator to protect themselves against the distraught caused by the reality of their own abuse suffered as a child (Minnaar, 2015). Identifying with a past perpetrator allows the child victim to understand the behavior the perpetrator wants them to display and helps the child to disarm the perpetrator, giving them a sense of control over the situation (McCartan et al., 2015; Johnson, 2019). Unfortunately, the internalization of this sense of control and rationalizing the behavior of the perpetrator is the same internal process the once child victim of abuse uses to justify their actions as a perpetrator of child rape (McCartan et al., 2015; Johnson, 2019). Many perpetrators also reenact their own abusive scenarios in an effort to gain mastery and control over unresolved trauma memories (Ardino, 2012).

#### Theme 3: Substance Abuse

The third theme refers to the use of alcohol and drugs by the participants themselves as well as substance abuse by family members and the influence it had on them as children. The use of substances during rape perpetration was also a common theme. The sub-themes that emerged were history of alcohol use, history of drug use and intoxication during rape perpetration.

##### History of Alcohol and Drug Use

Some participants indicated that they used alcohol or drugs frequently. Some also reported selling drugs and being arrested for dealing in drugs:

*I was a 5-year-old, I still remember. I started smoking dagga. I stopped for 1 year, start* [then started] *again. I was selling drugs* (Participant 4).

*I was selling drugs. I was under the influence of alcohol and drugs, that made me do the case* [commit the rape] (Participant 13).

Many participants reported that their parents were alcohol or drug users and dealers:

*He* [father] *would be drinking heavily* (Participant 3).

*My mother was drinking but stopped drinking. She would send me to buy her* [alcohol] (Participant 5).

*He* [father] *became an alcoholic in a way that he never done* [did] *anything for me* (Participant 6).

*My father was drinking alcohol. My mother brewed and sell* [sold] *traditional beer* (Participant 16).

*My father was arrested* [for] *selling drugs and dagga* (Participant 5).

##### Intoxication During Rape Perpetration

Some participants expressed that intoxication was the reason why they committed the rape and are currently incarcerated. These participants believed that intoxication led them to commit rape:

*I think it was alcohol and failing to control my feelings due to alcohol because it happened when I was drunk* (Participant 6).

*I was under* [the] *influence of alcohol and drugs, that made me do the case* [rape] (Participant 8).

*I didn’t believe it myself, cause when it* [rape] *happened I was under the influence completely, I drink* [drank] *a bottle of brandy plus I smoke* [smoked] *so much weed before* [the rape], *I was beyond intoxicated. I was out* (Participant 2).

Historically alcohol and drug abuse are significant health concerns in South Africa (Pienaar and Savic, 2016). Alcohol dependence and early onset of alcohol abuse (in childhood or adolescence) is strongly associated with childhood trauma exposure (Moustafa et al., 2021). Prolonged alcohol abuse may result in a blunted response to trauma and an impaired ability to recognize antisocial behavior as deviant and potentially harmful to others (Brewer-Smyth et al., 2004; Brewer-Smyth and Burgess, 2019). This may explain the relationship between alcohol abuse and an increased risk for rape perpetration, especially in cases where rape perpetration is explained away by alcohol use (Ramsoomar et al., 2021).

In the past 15 years there has been a major outbreak of methamphetamine use in South Africa (locally known as Tik) which is often accompanied by gangsterism and is rife in low socioeconomic communities characterized by limited policing and addiction care services (Asante and Lentoor, 2017). Methamphetamine use is associated with increased involvement in criminal activities, a high risk for arrest, increased sex drive and aggressive and impulsive behavior, making is a catalyst for rape perpetration (Watt et al., 2017; Stockman et al., 2021). Methamphetamine use is also more prevalent among survivors of childhood sexual abuse and is associated with commercialization of sex and increased HIV risk behavior (Meade et al., 2012; Watt et al., 2017). Given the high prevalence of childhood trauma reported in this study it is not surprising that participants also reported early onset of alcohol and drug use and being under the influence of substances when the rape occurred.

#### Theme 4: Gender Roles and Avoiding Responsibility

Traditional gender roles and violation of these roles are often used to justify rape and avoid responsibility for criminal behavior. Women are blamed for provoking men while violence displayed by men is viewed as a legitimized masculine trait. Traditional views on gender roles and methods of avoiding responsibility emerged in the sub-themes and included victim blaming, rape as male prerogative, transaction sex, being framed or “set-up” and ignoring an ancestral call.

##### Victim Blaming

A few of the participants indicated that the victims were the ones to initiate sexual contact and they were to blame for rape convictions.

S*he was all over me touching me, I realized something was happening. When I turned, she was close to me, we kissed and fell on to the matrass, we kissed for a long time* (Participant 6).

*She came in, she told me she like* [likes] *me, she came on top of me. I didn’t go to her, that’s what happened* (Participant 10).

One participant indicated that he was not to blame for rape since the victim was not a virgin:

*They called, inform me that I’m responsible for damages. I said, we can’t speak over the phone, but I didn’t take her virginity*…*I realized that I might be punished for other people’s sins* (Participant 5).

##### Rape as Male Prerogative

Two participants showed a lack of understanding regarding the rape of a spouse because they believed that a husband or long-term intimate partner is entitled to sex at any time with his partner and the partner cannot refuse him sex because they are married or in a long-term relationship:

*The female I’m married too, I can do anything to her, there’s something like that in isiZulu* [local language and cultural group] *saying that my wife can’t give me directives, I give directives to her. Whenever if I want to sleep with her, I should. I think others still have that thinking that a woman should listen to me as a man. Even though she is not in a mood that day, but she will do it. I think that is also part of it* (Participant 3).

*It can happen that you think something is right, but it is not right but as you are educated you realize that it was wrong, like if you knew that, if you are married you can sleep with a woman anytime because you paid for her, but after being educated you realize that it was wrong. I also learned, I thought if you are dating, I can come in anyhow without knocking when she is bathing while she may be uncomfortable, I learnt that. I thought that you can just enter because you are dating* (Participant 16).

##### Transactional Sex

One participant indicated that buying a woman something (in this case alcohol) entitled him to sex:

*We had already talked with my friends that since we bought them alcohol, we were going to have sex with them* (Participant 13).

##### Being Framed or “Set-Up”

Participants indicated that the victims framed them for rape for financial gain:

*I got a very rich sister*…*she’s very rich, so they wanted money, so they set me up with their niece* (Participant 4).

*The mother of the child wanted me to find a job* [for her]. *I told her* [name of company omitted] *doesn’t hire women, she wanted me to find a job at* [for] *cleaning* (Participant 14).

Participants indicated that they were framed or “set-up” by the victim due to pressure from the victim’s family or on behalf of someone else who was the real rape perpetrator:

*She did what she did to sort her family problems, because her family told her that if she does not arrest me* [get me arrested], *they would* [will] *disown her* (Participant 17).

*To be honest, there is nothing that can make me rape. She did it to protect her uncle because it was obvious, I was arresting* [arrested on behalf of] *her uncle*. Participant 9).

Another participant reported that his wife framed him for the rape of her child to prevent him from informing both his family and the wife’s family of her infidelities.

*She is making sure that I don’t go to her family and decided to frame me with rape of her child* (Participant 9).

##### Ignoring an Ancestral Call

One participant claimed that he was being punished for rape because he ignored his ancestral calling:

*I am supposed to be in prison because I don’t* [didn’t] *listen to my ancestors, I ignored their call when they need* [needed] *something. Now they are punishing me with prison* (Participant 9).

Many participants avoided taking responsibility for their actions and rationalized the act of rape by externalizing responsibility and rationalizing behavior by blaming it on the victim, gender stereotypes, transactional sex and being set-up by others. These findings correspond with prior findings where avoiding responsibility has been identified as a key characteristic of individuals displaying antisocial behavior (Pronyk et al., 2006; Selepe et al., 2020). Along with the aforementioned factors, substance use (as discussed in the previous section) is also commonly used as a way to avoid responsibility and a direct link between traditional views on gender roles and an increased risk for rape perpetration has been reported (Johnson, 2019; Selepe et al., 2020). The fact that only four out of the eighteen participants pleaded guilty further illustrates the avoidance of responsibility and potentially the lack of remorse associated with antisocial behavior (Russell and Hand, 2017).

#### Theme 6: Recidivism

Many participants reported having been involved in criminal activities before being convicted of rape. A few of the participants reported that they had been arrested for rape prior to their current conviction.

*Yes*, [arrested for rape for] *my sister’s kids, my nephews I was charged for my nephews* (Participant 1).

*I was accused for rape and murder, and convicted on rape and assault, so he gave me 2 years for assault and give* [gave] *me 7 years for rape, that’s 9 years* (Participant 4).

Other participants indicated that they had been accused/arrested for other criminal activities before their current rape conviction.

*I’ve been arrested for drugs, been arrested for stealing cars now I’m arrested for rape* (Participant 2).

*I was accused once; I was arrested for it and I was not convicted. I was arrested for attempted robbery before and convicted for 5 years* (Participant 8).

*I was arrested for a house breaking and sentenced 9 months* (Participant 16).

Recidivism was a common theme with participants reporting prior convictions for rape, physical assault, theft, robbery and drug use. Childhood trauma has been identified as a key driver of criminal behavior and multiple conviction, especially if the individual displays antisocial personality traits (Brewer-Smyth and Burgess, 2019; Frazier et al., 2019; Johnson, 2019). Recidivism is also more common when the perpetrator was raised in a context where criminal and violent behavior was common and was condoned by family members, community members or friends (Minnaar, 2015). It is evident from the findings in this study that many participants were exposed to criminal behavior as children since most participants reported the arrest of a parent, sibling or neighbor and this may contribute to a smaller chance of sustained successful rehabilitation following imprisonment and completion of rehabilitation programmes (Naidoo and Van Hout, 2022).

## Conclusion

In conclusion, in this study it was found that all participants were exposed to at least one form of childhood trauma. The psychological consequences of childhood trauma likely underlie the development of antisocial criminal behavior (Frazier et al., 2019; Johnson, 2019). Many of the key characteristics of antisocial behavior (e.g., lack of empathy, shame and guilt; inability to take responsibility for actions; impulsive, erratic and violent behavior; lack of meaningful intimate relationships; and a lack of morality) were observed in this study and are illustrated by the narratives of the participants (Barnett and Mann, 2013). Rape perpetration was further perpetuated by substance abuse and traditional gender roles. Many participants avoided taking responsibility for the rape and were known to the criminal system with prior offenses being a regular occurrence. The findings of the study demonstrate the complicated personality dynamics involved in the cycle of abuse and the evolution of criminal behavior, starting as a victim and ending as a perpetrator (Plummer and Cossins, 2018).

### Clinical, Legal and Policy Implications

Since our findings indicate that childhood trauma is the most common underlying experience of convicted rapists, it is worth exploring the current violence prevention strategies in South Africa and to identify room to improve and develop these strategies. Firstly, corporal punishment (including all forms of physical force, pain, discomfort and humiliation as a means to correct inappropriate behavior) has been linked to an increased risk for antisocial and criminal behavior mediated by feelings of fear, guilt and shame and socially inappropriate ways of dealing with danger and displaced anger (Mulvaney and Mebert, 2007; Morris and Gibson, 2011). Although corporal punishment has been outlawed since 1996 in South African schools and in the judicial services, it was only till recently (2019) that corporal punishment in the household was outlawed and to date there is limited effort to enforce the law (Children’s Amendment Bill, 2020). This is likely a reflection of the historical authoritarian approaches to discipline and the still prevalent general acceptance and practice of corporal punishment as a means to correcting problematic behavior (Ngubane, 2019; Mahlangu et al., 2021). The use of corporal punishment is further perpetuated by community violence exposure, domestic violence exposure, low socio-economic status and a lack of parental affection and love (Mahlangu et al., 2021).

A recent review of the literature on successful interventions for prevention of violence against children in the Global South suggest that interventions should be aimed at multiple stakeholders, e.g., schools (educator training on positive feedback approaches to discipline and psychosocial support to improve behavior and disclosure of abuse and neglect); parents (positive parenting skills improving the parent-child relationship); learners (psychoeducation); and non-governmental organizations, community leaders and community members (community activism, economic empowerment) (El-Khodary and Samara, 2020). Age and gender specific interventions collectively targeted at the individual, interpersonal, community and society level are also needed (Mathews et al., 2021). At the adolescent and young adult level prevention of interpersonal violence is key to breaking the cycle of abuse and preventing the exposure of future generations to childhood trauma in the household and community (El-Khodary and Samara, 2020; Mathews et al., 2021). In resource limited settings, these interventions should ideally be group-based peer lead interventions that focus on gender norms, healthy relationships and fostering positive coping styles and resilience (Minhyo Cho and Park, 2015; Kaljee et al., 2017). While multiple interventions targeted to multiple groups and on multiple levels may be resource intensive, it does have the potential to be cost-effective if integrated within existing routine service delivery (Bourey et al., 2015; Ferrari et al., 2022).

In general, not much is known about recidivism in South Africa and increasing research efforts are needed in this area, especially given that violence exposure, growing up in a disorganized family structure and substance use are prevalent social problems and are factors contributing to rape perpetration and recidivism (Gantana et al., 2015). Some efforts have been made by the South African judicial system to emphasize the severity of rape in the past 10–20 years, for example (1) the duration of incarceration has increased significantly (Thompson and Simmonds, 2012); (2) since 2007 a register of sexual offenders has been kept although it is only accessible to employers seeking to employ people who work with children and disable people (Sexual Offences and Related Matters Amendment Act 32 of 2007, 2007); and (3) since January 2022 all incarcerated rape perpetrators are obligated to provide a DNA samples (if not provided previously) and may not refuse the procedure (Criminal Law (Forensic Pocedures) Amendment Bill, 2022). It is envisioned that storing DNA profiles of rape perpetrators on the National Forensics DNA database will aid in solving cold cases, increase convictions and deter recidivism in the future (Criminal Law (Forensic Pocedures) Amendment Bill, 2022).

The role of current practices around rehabilitation in South African prisons and their potential for reducing recidivism has been questioned, especially in the context of prison overcrowding, prison gang activity and resource constraints (Cameron, 2020). Rehabilitation is further constrained by poor integration into the community following completion of a sentence, due to a lack of employment opportunities, high levels of poverty and low levels of education (Khwela, 2014). Correctional services offer a rehabilitation program that is not compulsory at the point of entry into the system but is rather completed toward the exit point and is an obligation for patrol eligibility (Singh, 2014; Murhula and Singh, 2019). The programme follows a “one size fits all” approach and covers broad categories such as general life skills, health and mental health education, skills development and moral growth (Murhula and Singh, 2019). A few studies investigated rehabilitation programmes in correctional services in South Africa have reported that they lack an individualized targeted approach to discovering the factors that contribute to and sustain criminal behavior (Herbig and Hesselink, 2012). They have also reported that there is an emphasis on completing the rehabilitation programme rather that investigating the behavioral effect/outcome of the programme as a measure of its success (Murhula and Singh, 2019). Rehabilitation approaches such as victim-offender mediation, understanding the impact of individual crimes on the community and enhancing the wellbeing and capabilities of perpetrators have been suggested as complementary measures for successful rehabilitation (Sherman et al., 2015). However, these approaches are costly and may not be a realistic rehabilitation approach given widescale resource constrains in South Africa (Gantana et al., 2015; Murhula and Singh, 2019). Closer investigation into the causes and contributors to rape perpetration and recidivism is needed to proactively prevent rape perpetration and may be more cost-effective and successful than retrospective rehabilitation programmes.

### Strengths and Limitations

The findings are specific to this study and may not be generalizable to other similar settings. The study is a qualitative study which may informs future complementary quantitative research on the factors that influence sexual violence perpetration in South African males. The study design allowed in-depth exploration of the life experiences of perpetrators of rape that may have contributed to criminal behavior. The translation and transcription of interviews were completed by researchers and not a professional translator which may have resulted in some linguistic nuances being lost.

## Data Availability Statement

The raw data supporting the conclusions of this article will be made available by the authors, without undue reservation.

## Ethics Statement

The study was approved by the Biomedical Resource Ethics Committee (BREC) of the University of KwaZulu-Natal (BE 129/19), and the Research Ethics Committee of the Department of Correctional Service, South Africa. The participants provided their written informed consent to participate in this study.

## Author Contributions

LN and LQ conceptualized and designed the study. LN collected, translated and transcribed data, and wrote the manuscript draft. LN, LQ, RM, and AW analyzed data. JN wrote discussion. LN, LQ, RM, AW, and JN read and revised the manuscript. All authors agreed to the submitted version.

## Author Disclaimer

The content hereof is the sole responsibility of the authors and do not necessarily represent the official views of the SAMRC or the funders.

## Conflict of Interest

The authors declare that the research was conducted in the absence of any commercial or financial relationships that could be construed as a potential conflict of interest.

## Publisher’s Note

All claims expressed in this article are solely those of the authors and do not necessarily represent those of their affiliated organizations, or those of the publisher, the editors and the reviewers. Any product that may be evaluated in this article, or claim that may be made by its manufacturer, is not guaranteed or endorsed by the publisher.

## Footnotes

1 Heavy leather/plastic whip.
